# The Typical “Cracked Dry Mud” Ultrasound Pattern of Proliferative Myositis

**DOI:** 10.5334/jbsr.1947

**Published:** 2019-10-11

**Authors:** Brice Dion, Bruno Coulier, Stéphane Van den Broeck

**Affiliations:** 1 Clinique Saint-Luc, Bouge, BE

**Keywords:** Proliferative myosistis, pseudosarcomatous, ultrasonography, MRI, Pectoralis major

## Abstract

**Teaching point:** The typical “cracked dry mud” pattern on ultrasound may help avoiding biopsy for proliferative myositis which is a self-resolving benign process.

## Case report

**Figure 1:**
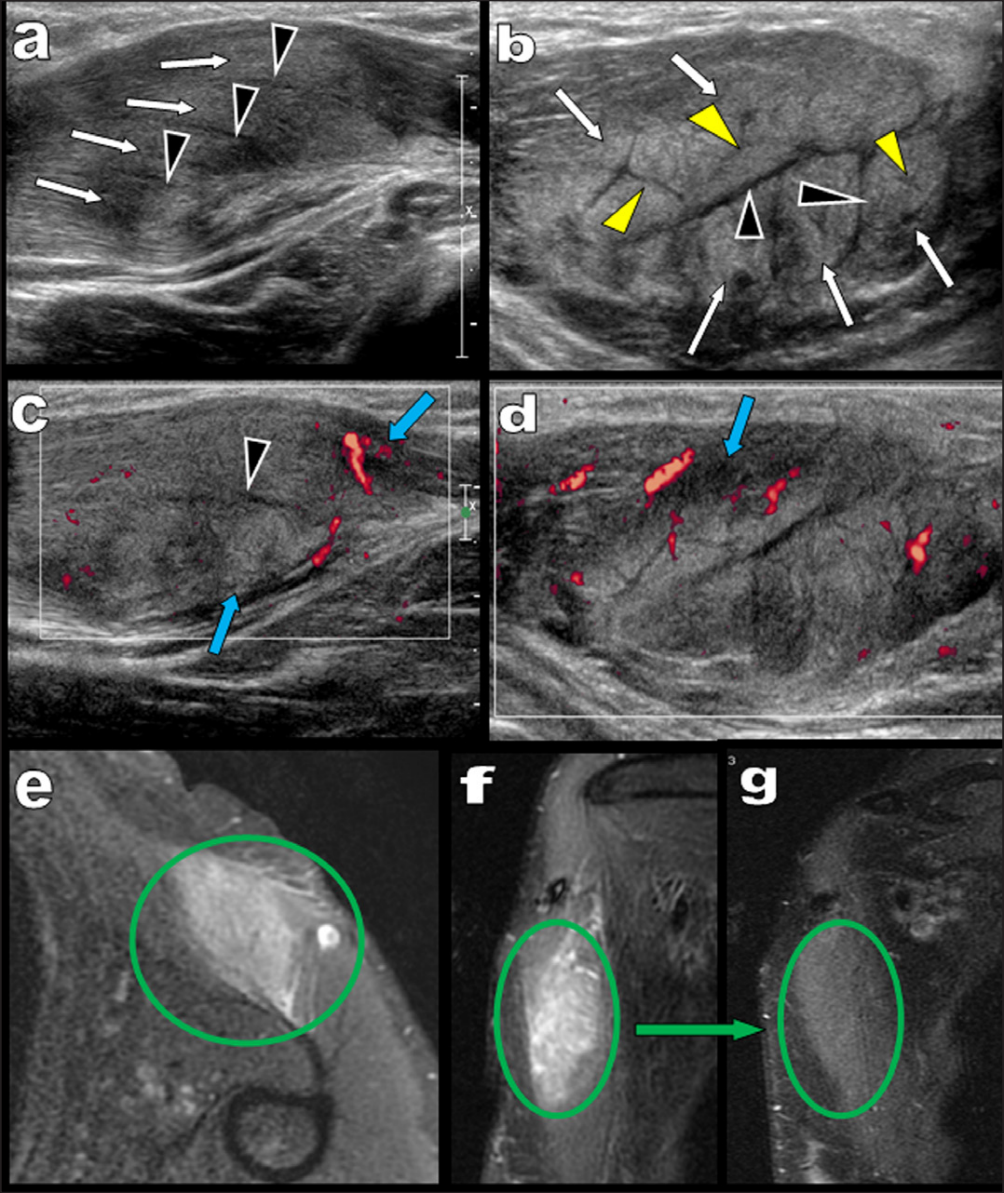


**Figure 2:**
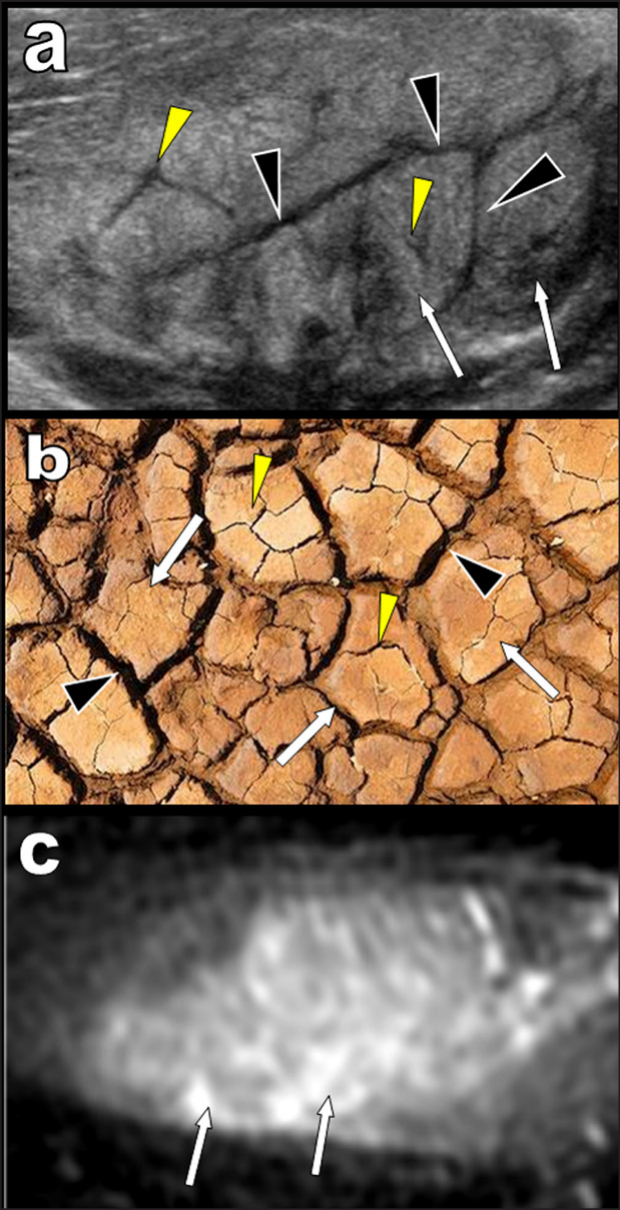


Muscular ultrasound (US) was required for a 53-year-old woman presenting with a rapidly growing firm and painless mass developing near the humeral insertion of the left pectoralis major. Longitudinal US (Figure 1a and 1c) showed intact but swollen hyperechoic muscle fascicles (white arrows) dissociated by vascularized hypoechoic bands (black arrowheads). Transverse US (Figures 1b, 1d and 2a) identified a subtle additional network of fine hypoechoic bands (yellow arrowheads) within the swollen muscle fascicles. Vascularized hypoechoic bands were also visible in the perimuscular fascia (blue arrows on Figure 1c and 1d). The typical “dry cracked mud” pattern of proliferative myositis (PM) was recognized (Figure 2b) and confirmed by fat suppressed T2 weighted MRI imaging (Figure 1e) showing focal hyperintense muscular enlargement. Magnetic resonance imaging (MRI) views perpendicular to the long axis of the muscle also found intact muscular fascicles within the area of hyperintensity (Figures 1f and 2c). The patient was very reluctant toward biopsy, so careful watch-and-wait follow-up was proposed. Control MRI performed after eight weeks confirmed complete healing (Figure 1g).

## Comment

PM is a rare self-limiting and rapidly growing worrisome pseudosarcoma process of skeletal muscles affecting middle-aged patients [1]. Nevertheless, PM is benign and generally spontaneously regresses and/or completely resolves without recurrence. PM belongs to the group of benign pseudo-sarcomatous processes including proliferative fasciitis, nodular fasciitis and intravascular fasciitis. The mostly painless – and rarely painful – process has a predilection for the trunk, head and extremities (especially the shoulder area). Trauma, mechanical injury or ischemia have been suggested as predisposing factors. Because PM mostly disappears spontaneously, no specific treatment is recommended following diagnosis.

PM diffusely involves the muscular connective stromal network (endomysium, perimysium and epimysium) with respect to the muscular fibres. It is constituted by a poorly demarcated network of myofibroblasts – accompanied by large basophilic giant cells resembling ganglion cells – infiltrating and dissociating the muscle fibers [1]. The cells are intimately intermixed with various amounts of collagen and mucoid material rich in hyaluronic acid.

Computed tomography lacks of any specificity but MRI may show more diagnostic features. T2-weighted MR images, STIR sequences and post-contrast T1-weighted images typically demonstrate a moderate to marked hyperintense and enhancing soft-tissue infiltrating mass. The geometrical network of hypointense bundles of muscle fibres surrounded by hyperintense inflammatory tissue may be seen on views perpendicular to the muscular fascicles.

US has a high spatial resolution and unlimited capabilities of multiaxial imaging facilitating optimal exploration of superficial muscles along their longitudinal or transverse axis. US is usually able to recognize the typical network of peri-, inter- and intra-fascicular inflammatory infiltration of PM with respect of the muscle fibres. The very typical “dry cracked mud” US pattern found on transverse views is unequivocally suggestive of PM [1].

As illustrated in our case, the robustness of US diagnosis of PM could allow a watch-and-wait option as an alternative method of follow-up, avoiding unnecessary biopsy and/or potentially mutilating surgery. Fine-needle biopsy would be reserved only for doubtful cases.
